# Neuroendocrine carcinoma of gallbladder: a case series and literature review

**DOI:** 10.1186/s40001-019-0363-z

**Published:** 2019-02-04

**Authors:** Wei Liu, Weijie Chen, Jiemin Chen, Tao Hong, Binglu Li, Qiang Qu, Xiaodong He

**Affiliations:** Department of Surgery, Peking Union Medical College Hospital, Chinese Academy of Medical Sciences, Shuaifuyuan 1#, Beijing, 100730 People’s Republic of China

**Keywords:** Neuroendocrine carcinoma, Gallbladder, Neuroendocrine tumor, Surgery

## Abstract

**Background:**

Neuroendocrine carcinoma (NEC) of gallbladder is a rare tumor. The clinical manifestation, treatment, and prognosis of gallbladder NEC are rarely reported.

**Case presentation:**

Eight gallbladder NEC patients were admitted into our hospital. The major complaint was right upper quadrant pain. Two patients underwent a radical resection of gallbladder and liver quadrate lobe. One of them underwent chemotherapies and had no recurrence of NEC during a 25-month followed-up period. The other patient did not undergo chemotherapies, and the NEC recurred in the patient 15 months afterwards. Two patients underwent a radical resection of gallbladder. One of them underwent chemotherapies and had an NEC recurrence 12 months afterwards. The other patient did not undergo chemotherapies and died due to the NEC recurrence 5 months after surgery. Three patients underwent a laparoscopic cholecystectomy and pathologic result showed gallbladder NEC. They did not undergo further treatment and no NEC recurrence was found. One patient underwent tumor biopsy and died due to obstructive jaundice 3 months afterwards. Pathologic results showed that all cases had positive chromogranin A and synaptophysin staining.

**Conclusions:**

Gallbladder NEC showed no noticeably specific features, and the diagnosis relied on the pathological and immunohistochemistrical results. For T1N0M0 gallbladder NEC, cholecystectomy might be enough. For patients in a late stage, the management of combined therapies might be optimal.

## Background

Neuroendocrine tumor (NET) is a rare neoplasm with the incidence of about 5.25 per 100,000 [1]. It has been found in many organs, such as gastrointestinal tract, lungs, and thyroid. Anus, jejunum, ileum, and pancreas are commonly involved in gastrointestinal tract, while gallbladder involvement is rarely reported [2]. According to a survey from 1973 to 2005 by “Surveillance, Epidemiology and End Result (SEER)” program hosted by National Cancer Institute, the incidence of gallbladder NET was less than 0.74/100,000 [1]. As a kind of poorly differentiated NET, neuroendocrine carcinoma (NEC) of gallbladder is even less. It occupied only 0.2% of all gastrointestinal NECs [1]. Due to the low morbidity, the clinical manifestation, management, and prognosis were rarely described. Aimed to investigate the characteristics of gallbladder NEC, we reviewed the cases we treated and the previous published articles.

## Case presentation

There are 8 gallbladder NEC patients, 2 male and 6 female, admitted into our hospital (Table 1). The main complaint (6 patients) was the right upper quadrant pain. One patient complained weight loss, and another one had no complains. He was admitted for an incidental finding of gallbladder tumor during a routine health checkup. No carcinoid syndrome-related symptoms such as diarrhea, flushing, edema, or wheezing were complained. The period from initial onset of symptoms to admission varied from 2 weeks to 3 years.

**Table 1:** Clinical features of eight cases of neuroendocrine carcinoma

No.	Age (years)	Gender	With fever	With right upper quadrant pain	With jaundice	Abdominal mass	Weight loss	With flushing	With diarrhea	With edema
1	57	Male	No	No	No	No	No	No	No	No
2	70	Female	No	No	No	No	Yes	No	No	No
3	56	Female	No	Yes	No	No	No	No	No	No
4	63	Female	Yes	Yes	Yes	No	No	No	No	No
5	47	Female	No	Yes	Yes	No	No	No	No	No
6	69	Female	No	Yes	No	No	No	No	No	No
7	57	Male	No	Yes	No	No	No	No	No	No
8	46	Female	No	Yes	No	No	No	No	No	No

**Fig. 1:**
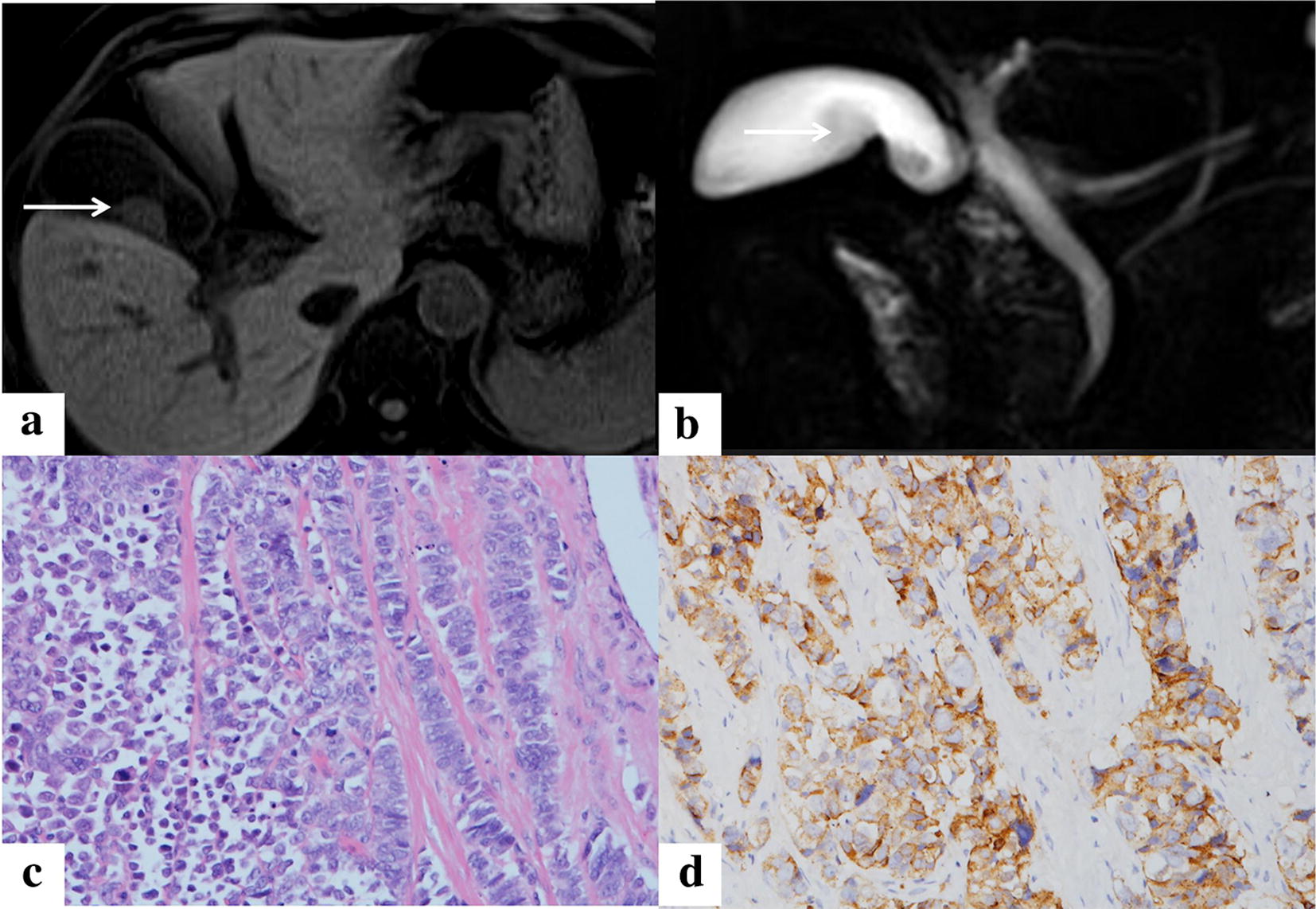
MRI and immunohistochemistry of gallbladder neuroendocrine carcinoma. **a**, **b** MRI image of gallbladder. The arrow showing a mass in the gallbladder. **c** HE staining of carcinoma tissues form gallbladder (×200). **d** CgA staining shows CgA positive cancer cells (×200)

All medical imaging examinations such as ultrasonography, CT, or MRI-detected masses in gallbladder and thickening of gallbladder were performed (Fig. 1). Most NECs (seven cases) were located in the gallbladder body. MRI showed that NEC in two cases had penetrated gallbladder wall and invaded liver. Gallbladder stones were found in one patient.

Two patients underwent a radical resection of gallbladder and liver quadrate lobe, because liver invasion was found pre-operationally (Table 2). One of them underwent chemotherapies with four cycles of etoposide plus cisplatin and subsequent two cycles of gemcitabine and had no recurrence of NEC during a 25-month followed-up period. The other case did not undergo chemotherapies, and NEC recurred in the patient 15 months afterwards. Two patients underwent a radical resection of gallbladder. One of them underwent chemotherapies with one cycle of irinotecan and cisplatin and had an NEC recurrence 12 months afterwards. While the other patient did not undergo chemotherapies and died due to the NEC recurrence 5 months after surgery, three patients underwent a laparoscopic cholecystectomy and pathologic result showed gallbladder NEC, and they did not undergo further treatment and no NEC recurrence was found. One patient underwent tumor biopsy, because lymph node metastasis was observed. The patient died of obstructive jaundice 3 months after biopsy.

**Table 2:** Treatment and prognosis of neuroendocrine carcinoma

No.	Surgery	Margin	Chemotherapy	Radiotherapy	Follow-up (months)	Prognosis
1	Radical dissection of gallbladder and liver quadrate lobe	R1	No	No	15	Recurrence
2	Radical dissection of gallbladder and liver quadrate lobe	R2	4 cycles of EP + 2 cycles of gemcitabine	No	25	No recurrence
3	Radical dissection of gallbladder	R3	NA	NA	5	Death
4	Radical dissection of gallbladder	R2	1 cycle of irinotecan and cisplatin	No	12	Recurrence
5	Gallbladder mass biopsy	NA	No	No	3	Death
6	Laparoscopic cholecystectomy	NA	NA	NA	29	No Recurrence
7	Laparoscopic cholecystectomy	NA	NA	NA	29	No Recurrence
8	Laparoscopic cholecystectomy	NA	NA	NA	45	No Recurrence

*EP* etoposide and cisplatin, *NA* not available

Pathologic results showed that two cases were small cell NECs, and two cases were large cell NECs (Table 3). All the cases of NEC showed positive Ki-67 staining in immunohistochemistry, with a range of 12% to 85%. Chromogranin A and synaptophysin were also positive in all cases.

**Table 3:** Pathological features of eight neuroendocrine carcinoma cases

No.	Location	Size (cm)	Type	TNM grade	Ki-67 (%)	Immunohistochemistry	With adenoma	With gallstone
1	Body	3.4 × 1.7	NA	T_4_N_0_M_0_	40	CEA (+), CDX2 (+), CGA (+), syn (+), CAM5.2 (+), CK19 (partly +), p53 (+)	Yes	No
2	Body	3.1 × 1.6	NA	T_4_N_0_M_0_	12–60	CK7 (−), CD56 (−), CGA (+), syn (+), EMA (+)	Yes	No
3	Body	2.1 × 1.0	NA	T_3_N_1_M_0_	70	CD3 (−), CK20 (−), CD56 (+), CGA (+), syn (+), alk-sp8 (−), BCL (−), CD10 (−), CD30 (−), MUM-1 (−)	No	No
4	Body	2.4 × 1.8	Large cell	T_3_N_0_M_0_	85	CDX2 (−), CK7 (+), CK20 (−), CGA (+), syn (+)	Yes	No
5	Body	2.7 × 2.2	Small cell	T_3_N_2_M_0_	80	CD3 (−), CK20 (−), CD56 (−), CGA (+), syn (+), s100 (−), vimentin (−)	No	No
6	Fundus	1.9 × 0.9	Small cell	T_1_N_0_M_0_	80	CD3 (−), CK20 (−), CGA (+), syn (+), MUC (−), P53 (−), SMA (−)	Yes	Yes
7	Body	1.7 × 1.4	Large cell	T_1_N_0_M_0_	40	CK19 (+), CGA (+), syn (+), CAM5.2 (+), CK20 (−), p53 (+)	Yes	No
8	Body	1.5 × 1.2	NA	T_1_N_0_M_0_	80	CK20 (−), CGA (+), syn (+), s100 (−), vimentin (−), CD3 (−)	No	No

*CD* cluster of differentiation antigen, *CDX2* caudal type homeobox transcription factor 2, *CEA* carcino embryonie antigen, *CGA* chromogranin A, *CK* cytokeratin, *EMA* epithelial membrane antigen, *syn* synaptophysin, *NA* not available

## Discussion

The classification and nomination of NET evolved considerably in the past decades. Previously, gastrointestinal NET was named carcinoid tumor, but the name had been criticized for misunderstanding of its possible malignancy. In 1995, Capella first gave the nomination of “neuroendocrine tumor”, which was widely accepted soon [3]. Subsequently, the 2000 WHO classification of gastrointestinal tract NET was established according to Capella classification. With considerations of tumor size, vessel and perineural invasion, proliferation activity, local invasion, lymph node, and distant metastases, NETs were separated into well-differentiated NET with benign behavior, well-differentiated NET with uncertain behavior, well-differentiated NEC, and poorly differentiated NEC. Nevertheless, the classification used a hybrid of grading and staging without independent assessments. Given that tumor grade and stage are considered as separated parameters with independent prognostic significance, the 2010 WHO classification establish a grading of G1–G3 and a staging of TNM scheme. Well-differentiated tumors were divided to G1 (low grade) and G2 (middle grade), and all poorly differentiated NETs (mitotic figures > 20/10 HPF and Ki-67 staining > 20% positive) were graded G3 (NEC).

The origin of gallbladder NEC is controversial. Many researchers propose that tumors come from the metaplasia of gallbladder epithelium and the inflammations might promote metaplastic changes to neuroendocrine cells, because most NECs accompany with cholelithiasis. Normally, no neuroendocrine cells exist in gallbladder; however, neuroendocrine cells do exist in gallbladder of many cholelithiasis patients. Sakamoto et al. found that epithelium metaplasia happened in 11.7% patients with cholelithiasis, 83.3% of them showed positive staining of chromogranin A, and 50.0% had positive staining of serotonin [4]. These two antigens are specifically expressed in neuroendocrine cells. Other researchers insist that NEC is transformed from gallbladder adenocarcinoma. Many gallbladder NECs are concomitant with adenocarcinoma, and a mutual conversion of NET and adenocarcinoma in gastrointestinal tract has been proved [5]. Thus, this hypothesis cannot be denied either.

The clinical manifestations of gallbladder NEC vary. No specific signs were reported. In general, the main complaint is the right upper quadrant discomfort, including pains with distention and tenderness. However, the pain is undistinguishable from cholelithiasis. Carcinoid syndrome is rarely reported in gallbladder NECs. Functional NETs are capable of secretion of peptides such as histamine and serotonin. Commonly, no symptom was reported in gastrointestinal tract NET patients because of first-pass effect of liver. Only in a few cases, these peptides were not totally degraded, leading to clinical manifestations such as distention, diarrhea, flushing, edema, and wheezing [2]. The carcinoid syndrome was reported in cases of giant gallbladder NEC, patients usually suffered from severe distention and diarrhea. No flushing or edema was reported.

Ultrasonography, CT and MRI could detect solid masses of gallbladder, although it is unable to distinguish from other types of gallbladder carcinoma. They could also show suspected metastatic site and lymph nodes, and remains helpful for the TNM staging and surgery plan. Currently, the confirmative diagnosis of gallbladder NEC relies on pathology result and immunohistochemistry staining. Common biomarkers of immunohistochemistry are Chromogranin A and synaptophysin, with positive rate of 91.9% and 84.8% [6]. In addition, pathological results determine the tumor grading and staging.

There is no consensus of treatment of gallbladder NEC. Surgical management remains a first-line consideration. For in situ and T1N0M0 tumor, cholecystectomy could be enough [7]. In our study, three cases underwent a laparoscopic cholecystectomy and no further treatment. No NEC recurrences were found during at least 29-month follow-up. A radical operation for a late stage tumors includes local lymph node dissection and metastatic site dissection. For patients with distant metastasis, surgical treatment remains controversial. In general, a local liver invasion requires early a radical dissection to improve life quality and decrease tumor-induced complications [8]. Since the malignancy grade of NEC is high, many patients are in a late stage when diagnosed. Surgery alone is not enough for treatment. Chemotherapy remains uncertain in management of NEC because of its low sensitivity. The platinum-based chemotherapy regimens according to guideline of lung small cell carcinoma were used in treatment of NEC, and achieved impressive responses. Besides, radiotherapy and endocrine therapy were also used in NEC management [9].

The prognosis of gallbladder NEC is very poor. Duffy et al. reported that the median survival of gallbladder NEC was 9.8 months, lower than the median survival of gallbladder carcinoma [5]. In our study, untreated patient died 3 months afterwards. Patients who underwent surgery and chemotherapy have a prolonged survival. Up to now, there is a case with no recurrence after 25-month follow-up after surgery and chemotherapy. Shimono et al. reported a case with no recurrence after 69-month follow-up after surgery and post-surgical chemotherapy [8]. It seems that management of combined therapies is the optimal treatment.
